# Galectin-1 Facilitates Macrophage Reprogramming and Resolution of Inflammation Through IFN-β

**DOI:** 10.3389/fphar.2020.00901

**Published:** 2020-06-17

**Authors:** Hiba Yaseen, Sergei Butenko, Irina Polishuk-Zotkin, Sagie Schif-Zuck, Juan Manuel Pérez-Sáez, Gabriel Adrian Rabinovich, Amiram Ariel

**Affiliations:** ^1^ Departments of Biology and Human Biology, University of Haifa, Haifa, Israel; ^2^ Laboratorio de Inmunopatología, Instituto de Biología y Medicina Experimental, Consejo Nacional de Investigaciones Científicas y Técnicas, Buenos Aires, Argentina; ^3^ Facultad de Ciencias Exactas y Naturales, Universidad de Buenos Aires, Buenos Aires, Argentina

**Keywords:** resolution of inflammation, macrophage reprogramming, galectin-1, interferon-β, proteinase 3

## Abstract

During the resolution of acute inflammation, macrophages undergo reprogramming from pro-inflammatory, to anti-inflammatory/reparative, and eventually to pro-resolving macrophages. Galectin-1 (Gal-1) is a *bona fide* pro-resolving lectin while interferon β (IFN-β) was recently shown to facilitate macrophage reprogramming and resolution of inflammation. In this study, we found Gal-1^null^ mice exhibit a hyperinflammatory phenotype during the resolution of zymosan A-induced peritonitis but not during the early inflammatory response. This phenotype was characterized by reduced macrophage numbers, increased secretion of pro-inflammatory cytokines, such as interleukin-12 (IL-12), and reduced secretion of anti-inflammatory cytokines, such as interleukin-10 (IL-10). In addition, we found a delayed expression of the pro-resolving enzyme 12/15-lipoxygenase in macrophages and heightened levels of the inflammatory protease proteinase-3 (PR3) in peritoneal fluids from Gal-1^null^ mice. Moreover, we observed sex-dependent differences in the inflammatory profile of Gal-1^null^ mice. Notably, we found that IFN-β levels were reduced in resolution-phase exudates from Gal-1^null^ mice. Administration of IFN-β *in vivo* or *ex vivo* treatment was able to rescue, at least in part, the hyperinflammatory profile of Gal-1^null^ mice. In particular, IFN-β recovered a subset of F4/80^+^GR-1^+^ macrophages, restored IL-12 and IL-10 secretion from macrophages to WT values and diminished abnormal peritoneal PR3 levels in Gal-1^null^ mice. In conclusion, our results revealed a new Gal-1-IFN-β axis that facilitates the resolution of inflammation and might restrain uncontrolled inflammatory disorders.

## Introduction

Inflammation is a beneficial host response to foreign challenges or tissue injury that, when resolved in an effective and timely manner, leads to the restoration of tissue homeostasis (Filep, 2013; Sugimoto et al., 2019). Nonetheless, prolonged inflammation ceases to be beneficial, and in turn, contributes to the pathogenesis of various chronic diseases (Nathan and Ding, 2010; Spite et al., 2014). Manifestations of localized chronic inflammation include impaired wound healing, tissue fibrosis, and organ dysfunction (Ueha et al., 2012; Ariel and Timor, 2013; Gieseck et al., 2018; Krzyszczyk et al., 2018).

The active resolution of a localized acute inflammation culminates in the elimination of short-lived neutrophils by resolution-phase macrophages and the return of leukocyte cell populations to homeostatic numbers (Ariel and Serhan, 2012; Ariel and Timor, 2013; Greenlee-Wacker, 2016; Dalli and Serhan, 2017; Elliott et al., 2017). One of the key mechanisms underlying the tightly orchestrated resolution of inflammation is macrophage reprogramming. Macrophages undergo conversion from type I (M1-like, pro-inflammatory) to type II (M2c-like, anti-inflammatory/deactivated) macrophages (Ariel and Serhan, 2012; Greenlee-Wacker, 2016; Elliott et al., 2017; Atri et al., 2018). The clearance of apoptotic neutrophils leads to the reprogramming of resolution-phase macrophages to a distinct satiated/pro-resolving phenotype that is highlighted by the loss of its phagocytic properties and the departure of the injury site (Ariel and Serhan, 2012; Greenlee-Wacker, 2016; Elliott et al., 2017). Type I macrophages secrete pro-inflammatory cytokines, such as tumor necrosis factor-α (TNF-α), interleukin-6 (IL-6), and IL-12, whereas type II macrophages secrete anti-inflammatory cytokines, such as transforming growth factor-β (TGF-β) and in some cases IL-10 (Ariel and Serhan, 2012; Greenlee-Wacker, 2016; Elliott et al., 2017; Atri et al., 2018). As compared with type II macrophages, pro-resolving macrophages show reduced expression of surface CD11b, iNOS, and arginase-1 (Schif-Zuck et al., 2011). These macrophages also express an enzyme that excels in producing pro-resolving lipid mediators (SPM), namely 12/15-lipoxygenase (12/15-LO) (Schif-Zuck et al., 2011; Ariel and Serhan, 2012; Ariel and Timor, 2013; Headland and Norling, 2015; Dalli and Serhan, 2017). Notably, eosinophils are also key cellular effectors in the resolution of inflammation (Yamada et al., 2011; Isobe et al., 2012).

Galectin-1 (Gal-1), which belongs to a family of β-galactoside-binding lectins, has been described as a modulator of a wide range of immune responses, including macrophage-mediated inflammation (Arthur et al., 2015; Sundblad et al., 2017). The immunomodulatory properties of intracellular or extracellular Gal-1 are ascribed to its ability to inhibit immune cell adhesion, and alter cell signaling and cytokine production under inflammatory conditions (Arthur et al., 2015; Sundblad et al., 2017). We previously reported that Gal-1 treatment *in vivo* and *in vitro* facilitates reprogramming towards CD11b^low^12/15-LO^+^ pro-resolving macrophages (Rostoker et al., 2013). Recently, we uncovered another macrophage-derived resolution-phase effector cytokine – IFN-β (Kumaran Satyanarayanan et al., 2019). IFN-β belongs to the family of type I interferons, which confer cellular resistance to viral infections. However, it also conveys other biological functions in macrophages and T cells, including immune suppression and reprogramming in autoimmune diseases and bacterial infections, respectively (Severa et al., 2015; Snell et al., 2017).

Activated neutrophils secrete a specific set of serine proteases that contribute to their ability to migrate through the basement membrane, to degrade the extracellular matrix, and to digest pathogens (Kettritz, 2016). One of these serine proteases is proteinase-3 (PR3), which can be either secreted or expressed at the neutrophil cell surface (Kettritz, 2016; Thieblemont et al., 2018). PR3 interferes with macrophage phenotype conversion, thereby inhibiting resolution of inflammation (Millet et al., 2015; Thieblemont et al., 2018).

Here, we examined whether Gal-1^null^ mice that undergo zymosan A-induced peritonitis would exhibit a hyperinflammatory phenotype, including increased PR3 levels. Indeed, we found that Gal-1^null^ mice displayed reduced macrophage numbers with defective reprogramming and CD11b expression, as well as increased PR3 levels. Moreover, our results showed that IFN-β was upregulated by Gal-1 and acted as its downstream effector that rescued Gal-1 deficiency.

## Materials and Methods

### Antibodies and Recombinant Proteins

The indicated reagents were obtained as follows: ELISA kits for mouse TNF-α, IL-10, IL-6, and IL-12 (Biolegend), and for mouse CCL2 and CCL5 (R&D systems); FITC-conjugated anti-mouse Gr-1, FITC-conjugated anti-mouse Ly6G, PE-conjugated anti-mouse F4/80, PerCP-conjugated anti-mouse CD11b, PB-conjugated anti-mouse Ly6C and PE/Cy7-conjugated anti-mouse TLR4 (Biolegend); APC-conjugated anti-mouse Tim4 (Miltenyi); Alx647-conjugated anti-mouse IgG, goat anti-mouse arginase-1, and rabbit anti-IFN-β antibodies (Abcam); rabbit anti-mouse 12/15-lipoxygenase antibody (Cayman Chemical); goat anti-mouse CD11b, goat anti-mouse β-actin and goat anti-PR3 antibodies (Santa Cruz Biotechnology); anti-p-STAT-1 and anti-p-STAT3 (Cell Signalling); anti-goat and anti-rabbit horseradish peroxidase-conjugated antibodies (Jackson ImmunResearch laboratories). Recombinant Gal-1 was generously provided by the laboratories of GR and Dr. Lichtenstein (Universidad de Buenos Aires, Buenos Aires, Argentina; Ben-Gurion University, Be'er Sheva, Israel); monoclonal anti-Gal-1 IgG, isotype IgG and polyclonal rabbit anti-Gal-1 antibody were obtained from the laboratory of GR, and used as described (Toscano et al., 2007; Stowell et al., 2008; Rostoker et al., 2013).

### Mice

Male and female C57BL/6 wild-type (WT) mice (7–8 weeks old) were purchased from Harlan Biotech. Gal-1^null^ (*lgals* ^-/-^) mice on a C57BL/6 background were obtained from Dr. Goldenberg, The Hebrew University of Jerusalem. Mice were bred and maintained under special pathogen-free conditions in the animal facility at the Faculty of Biology in the Technion (Israel Institute of Technology, Haifa, authorization no. IL-009-01-2010).

### Zymosan A-Induced Peritonitis

Mice were injected intraperitoneally (i.p.) with freshly-prepared zymosan A (1 mg/ml, 1 mg/25 g body weight, Sigma-Aldrich) in sterile PBS, or were kept unchallenged. In some experiments, recombinant IFN-β (250 ng in 1 ml of PBS, Biolegend) was injected i.p. 24 to 48 h post zymosan A injection. At 24, 48, 66, or 96 h post-zymosan A injection, mice were euthanized with CO_2_, and peritoneal exudates were collected by lavage with 5 ml of sterile saline. Exudate cells and supernatants were obtained by centrifugation for further analysis and experimentation.

### Isolation of Murine Peritoneal Macrophages

Cells were recovered from peritoneal exudates at the indicated time points following zymosan A challenge, Gal-1 or IFN-β treatment. Macrophages were labeled with PE-conjugated rat anti-F4/80 antibody and isolated using EasySep-PE selection magnetic beads according to the manufacturer's instructions (Stem-Cell Technologies).

### Cell Culture

Murine peritoneal macrophages were obtained from mice at indicated time points post peritonitis. The macrophages were cultured (1 × 10^6^ cells/0.5 ml) in RPMI 1640 (GIBCO), supplemented with 10% fetal bovine serum (FBS), 2 µM glutamine, 100 units/ml penicillin, and 100 µg/ml streptomycin. In some experiments, the cells were treated with recombinant Gal-1 (4–8 µg/ml), IFN-β (25 ng/ml), LPS (1 µg/ml, Sigma-Aldrich), or vehicle controls. Following incubation of 30 min for p-STAT1/3 analysis or 16 to 24 h for other analyses, the cells and the cell-free supernatants were collected. The cells were evaluated by Western blotting or flow cytometry, and cytokine/chemokine levels (TNF-α, IL-1β, IL-12, IL-6, IL-10, CCL2, or CCL5) in the supernatants were determined by standard ELISA according to the manufacturer's instructions (R&D systems, BioLegend).

### Flow Cytometry

For the determination of leukocyte subtypes and expression of surface markers, exudate cells were first blocked with anti-CD16 and anti-CD32 antibodies to prevent non-specific staining. The cells were stained with FITC-conjugated anti-mouse Gr-1 (0.5 µg/10^6^ cells), FITC-conjugated anti-mouse Ly6G (0.2 μg/10^6^ cells), PE-conjugated anti-mouse F4/80 (0.2 µg/10^6^ cells), PerCP-conjugated anti-mouse CD11b (0.2 µg/10^6^ cells), PB-conjugated anti-mouse Ly6C (0.2 μg/10^6^ cells), PE/Cy7-conjugated anti-mouse TLR-4 (0.5 μg/10^6^ cells), and APC-conjugated anti-mouse Tim4, CD206, or CD163 (0.3–0.5 μg/10^6^ cells). For intracellular staining of Gal-1, cells were blocked with anti-CD16/CD32 Ab (Biolegend) first, and then stained for surface markers and fixated with 4% PFA in 5% sucrose/PBS for 15 min at RT, followed by permeabilization with 0.01% tween-20 in 1% BSA in PBS for 20 min on ice. Then, the cells were incubated with mouse anti-Gal-1 (0.5 μg/10^6^ cells) or without primary antibody and then with Alx647 or Alx488-anti-mouse IgG (isotype control) for 20 min at RT, washed with 1% BSA in PBS. Stained cells were analyzed using FACSCalibur or FACSCantoII (BD Biosciences). Data analysis was performed using the FlowJo software.

### Western Blot

Cells were collected, centrifuged, washed with PBS, and lysed in RIPA buffer containing Protease Inhibitors Cocktail (PIC, Sigma-Aldrich) and phosphatase inhibitor (phosStop, Roche), diluted according to the manufacturer's instructions. Proteins from cell-free exudates were also recovered. Alternatively, peritoneal fluids were centrifuged, and cell-free supernatants were recovered. Samples were run by SDS-PAGE (10%) and transferred to PVDF membranes. The membranes were immunoblotted with primary antibodies against the indicated antigens (see above), followed by immunoblotting with matching secondary antibodies. The membranes were developed with EZ-ECL detection kit (Biological Industries) and analyzed using Luminescent Image Analyzer LAS-4000 (Fujifilm Corporation). Densitometry was performed using TotalLab TL100 (nonlinear dynamics) image analysis software.

### Zymography

The caseinolytic activity of peritoneal fluids was analyzed by zymography. Casein from Bovine Milk (0.5 mg/mL, Sigma-Aldrich) was added to 11% SDS polyacrylamide gels, and equal amounts of peritoneal fluids (20 μl per well) was run under non-reducing conditions at 4°C. Gels were washed with PBS containing 2.5% Triton X-100, followed by incubation overnight at 37°C in a zymogram development buffer (Bio-Rad). Then, gels were washed, stained with Coomassie blue, and washed again with a de-staining solution. The gels were analyzed using Luminescent Image Analyzer LAS-4000 (Fujifilm Corporation). The caseinolytic activity was visualized as clear bands against a blue background, with detection sometimes obscured by overlapping of non-reactive proteins. Densitometry was performed using TotalLab TL100 (nonlinear dynamics) image analysis software.

### Statistical Analysis

Experiments were performed at least 2 times, with 4 replicates of each data point. Results were analyzed by the two-tailed Student's *t*-test and Mann–Whitney U test for comparison of two groups, and one-way Kruskal–Wallis ANOVA test for comparison of more than two groups. Data are presented as mean ± SEM. Results were considered statistically significant when p < 0.05 (*), 0.01 (**), 0.001 (***).

## Results

### Gal-1 Is Expressed Primarily by Mature Macrophages During the Resolution of Inflammation

**Figure 1:**
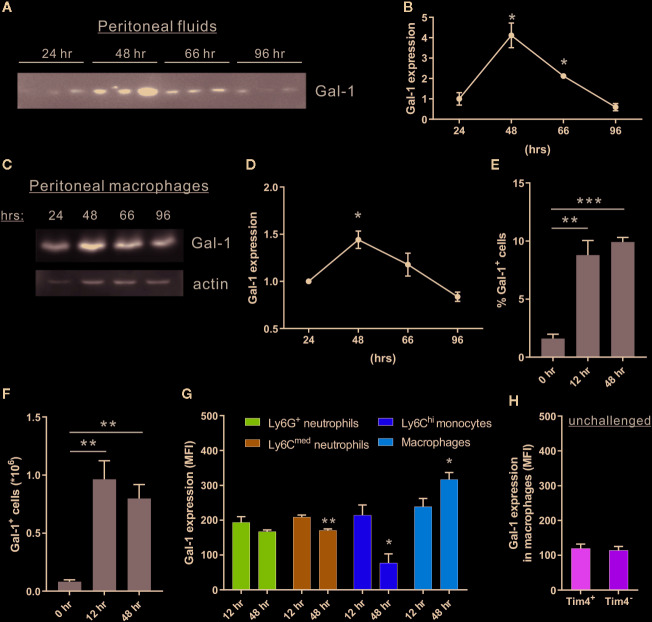
Peritoneal macrophages increase the expression of Gal-1 in response to acute inflammation. WT mice were injected intraperitoneally with zymosan A (1 mg/mouse in 1 ml of PBS) to initiate peritonitis. Peritoneal exudates were collected at 0, 24, 48, 66, and 96 h post peritonitis initiation (PPI). Cell-free fluids were separated, peritoneal cells were examined by flow cytometry (see **Figure S1C** for gating strategy), and macrophages were isolated from the cellular fraction using magnetic beads. **(A–D)** Peritoneal fluids **(A, B)** and lysates from macrophages **(C, D)** were run by SDS-PAGE, followed by immunoblotting for Gal-1 and actin as a loading control. **(A, C)** Representative blot images. **(B, D)** Gal-1 protein expression was quantified by densitometry, and expression relative to 24 h was calculated. Original blot images are presented in **Figure S7**. **(E–H)** Peritoneal leukocytes were enumerated, immunostained for the surface antigens Ly6G, Ly6C, F4/80, Tim4, and intracellular Gal-1, and analyzed by flow cytometry. **(E, F)** Gal-1^+^ leukocytes are presented in percentage or in total numbers/ml in peritoneal fluids, n = 4. **(G, H)** Intracellular Gal-1 expression (MFI) in the following leukocyte populations: Ly6G^+^F4/80^-^ neutrophils, Ly6C^med^F4/80^-^ neutrophils, Ly6C^hi^F4/80^lo^ monocytes, F4/80^+^Ly6C^-^ macrophages, F4/80^+^Tim4^-^ monocyte-derived resident macrophages and F4/80^+^Tim4^+^ yolk sac-originated resident macrophages, n = 4. Altogether, the data represent at least 2 independent experiments and are presented as mean ± SEM. Statistical analysis by two-tailed Student's *t*-test; *p < 0.05, **p < 0.01, ***p < 0.001.

Gal-1 administration in mice facilitates the resolution of acute inflammation (Rostoker et al., 2013; Sundblad et al., 2017). Using a spontaneously resolving model of zymosan A-induced peritonitis (Bannenberg et al., 2005; Cash et al., 2009), we detected upregulation of Gal-1 expression in peritoneal fluids and in isolated F4/80^+^ monocytes/macrophages, which peaked at 48 h post-peritonitis initiation (PPI), and declined afterwards (**Figures 1A–D** and **Supplementary Material, Figures S1A, B**). To identify the exact myeloid population that expresses Gal-1 we performed intracellular staining of Gal-1 in peritoneal cells that was analyzed using flow cytometry (see **Supplementary Material, Figure S1C** for gating strategy). Our results showed a relative increase in the percentage and number of Gal-1^+^ cells at 12 and 48 h PPI (**Figures 1E, F**), compared to unchallenged mice. Notably, the expression of Gal-1 reached maximal levels in F4/80^+^Ly6C^-^ mature macrophages at 48 h, whereas their Ly6C^hi^F4/80^lo^ precursors (Butenko et al., 2020) showed diminished expression of intracellular Gal-1 upon progression from the inflammatory (12 h) to the resolving (48 h) phases (**Figure 1G**). As expected from our Western blot results, both Tim4^+^ and Tim4^-^ peritoneal resident macrophages expressed the lowest levels of Gal-1 (**Figure 1H**). Ly6G^+^ neutrophils and Ly6C^med^F4/80^-^ cells (composed mostly of neutrophils (Butenko et al., 2020) showed very similar levels of expression of Gal-1 that did not change upon transition to the resolution phase (**Figure 1G**). The coordinated increase in Gal-1 levels in peritoneal fluids and F4/80^+^ macrophages is in accord with previous reports (Rostoker et al., 2013; Law et al., 2020), and suggests that macrophages are the major cell population responsible for Gal-1 secretion and peritoneal levels during the resolution of inflammation. The temporal changes in Gal-1 expression and release underscore the involvement of Gal-1 in the resolution of inflammation, which normally resolves within 48 to 96 h in the medium dose zymosan-induced peritonitis (Bannenberg et al., 2005; Cash et al., 2009; Schif-Zuck et al., 2011).

### Gal-1 Increases the Numbers of Peritoneal Neutrophils, Eosinophils, and Macrophages During the Resolution of Inflammation

**Figure 2:**
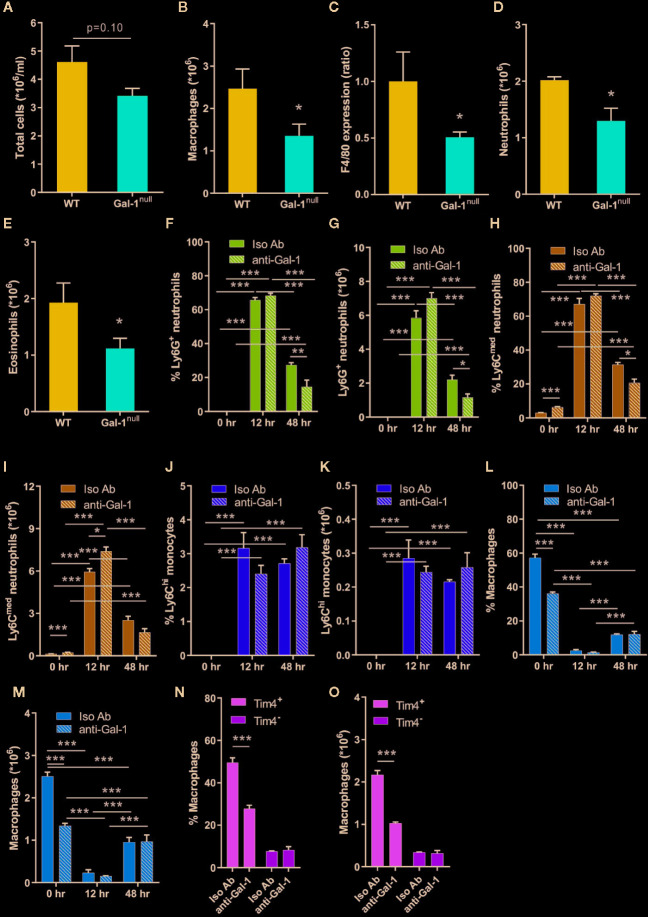
Gal-1 increases the numbers of peritoneal neutrophils, eosinophils, and macrophages. **(A–E)** Peritoneal exudates were collected from WT and Gal-1^null^ male mice 48 h PPI. Peritoneal leukocytes were enumerated, immunostained for Gr-1, F4/80, and CD11b, and analyzed by flow cytometry, n = 6–10. **(A)** The number of peritoneal leukocytes/ml in peritoneal fluids. **(B)** The total number of macrophages. **(C)** The ratio of F4/80 expression, based on MFI. **(D)** The total number of neutrophils. **(E)** The total number of eosinophils. **(F–O)** 12 to 24 h prior to peritoneal cell collection mice were administered with neutralizing anti-Gal-1 mAb (anti-Gal-1) or isotype IgG (Iso Ab). Peritoneal cells were collected from unchallenged mice, or at 12 or 48 h PPI. Upon recovery, peritoneal leukocytes were enumerated, immunostained for the surface antigens Ly6G, Ly6C, F4/80, and Tim4, and analyzed by flow cytometry. Eosinophils were gated out due to unspecific binding of 2^nd^ antibody to anti-Gal-1 mAb, which was performed concomitantly. Leukocytes are presented in percentage **(F, H, J, L, N)** or in total numbers/ml **(G, I, K, M, O)** in peritoneal fluids, n = 4. Measurements of the following leukocyte populations: Ly6G^+^F4/80^-^ neutrophils **(F, G)**, Ly6C^med^F4/80^-^ neutrophils **(H-I)**, Ly6C^hi^F4/80^-^ monocytes **(J, K)**, F4/80^+^Ly6C^-^ macrophages **(L, M)**, F4/80^+^Tim4^-^ monocyte-derived resident macrophages and F4/80^+^Tim4^+^ yolk sac-originated resident macrophages **(N, O)**. Altogether, the data represent at least two independent experiments and are presented as mean ± SEM. Statistical analysis by two-tailed Student's *t*-test; *p < 0.05, **p < 0.01, ***p < 0.001.

During the resolution phase of inflammation, there is a gradual increase in macrophage and eosinophil numbers, concomitantly with a decrease in the neutrophil numbers (Bratton and Henson, 2011; Ariel and Serhan, 2012; Ariel and Timor, 2013; Greenlee-Wacker, 2016). Moreover, Gal-1 is able to block neutrophil infiltration to inflamed sites during the onset phase (Rabinovich et al., 2000) and promote monocyte/macrophages migration (Malik et al., 2009; Gil et al., 2010). Curiously, some data indicates under non-inflammatory conditions Gal-1 can attract neutrophils to the peritoneum (Auvynet et al., 2013). Thus, we hypothesized that Gal-1 deficiency in mice would lead to altered leukocyte populations under inflammatory conditions. To test that hypothesis, we examined peritonitis in male Gal-1^null^ mice in comparison with their WT counterparts. In accordance with the peak in Gal-1 expression (**Figures 1A–D**), we detected a trend of decrease in total cell number in Gal-1^null^ males (**Figure 2A**). Flow cytometry of the isolated peritoneal cells revealed lower numbers of neutrophils, eosinophils, and macrophages in Gal-1^null^ males in comparison to their WT counterparts, together with reduced expression of the macrophage marker F4/80 (**Figures 2B–E**). To determine whether Gal-1 deficiency affects leukocyte numbers during the resolution phase exclusively, we treated WT male mice with neutralizing anti-Gal-1 antibodies (Toscano et al., 2007) or their isotype controls. Antibodies were administered 24 h before recovery of unchallanged peritoneal cells, or 12 or 24 h prior to recovery of cells at 12 or 48 h PPI, respectively. Our results (**Figures 2F–O**) determined Gal-1 neutralization did not affect neutrophil, monocyte or macrophage frequency or numbers at 12 h. However, Gal-1 neutralization did reduce neutrophil numbers (the same cell population was detected by two different staining protocols as either Ly6G^+^F4/80^-^ or Ly6C^med^F4/80^-^ cells) at 48 h PPI significantly. Curiously, the neutralization of Gal-1 in unchallenged mice resulted in a significant reduction in the frequency and numbers of F4/80^+^ macrophages (**Figures 2L, M**) due to a selective reduction in Tim4^+^ yolk sac-originated macrophages (**Figures 2N, O**), but did not affect the frequency or numbers of Ly6C^hi^F4/80^lo^ monocytes or Ly6C^-^F4/80^+^ macrophages [that are dominated by CD11b^hi^/CD206^hi^/CD163^hi^ M2-like cells (**Supplementary Material, Figure S1D**)], in comparison to isotype control treatment. Of Interest, intracellular Gal-1 expression in monocyte-derived macrophages, but not neutrophils, monocytes or resident macrophages, was reduced by neutralization of extracellular Gal-1 at either 12 or 48 h (**Supplementary Material, Figures S2A–E**). These findings suggest Gal-1 promotes its own expression by macrophages, and that the residual expression of Gal-1 in macrophages is sufficient to maintain their numbers during the resolution phase. Thus, macrophage-expressed Gal-1 seems to play an essential role in controlling leukocyte numbers during the resolution of inflammation.

The resolution of inflammation shows well-established sex-dependent differences in its kinetics (Wang et al., 2012). Therefore, we examined the impact of Gal-1 deficiency on leukocyte numbers at 48 h PPI in females. In contrast to our observations in males, Gal-1^null^ females did not show a decrease in leukocyte populations, except for a reduction in eosinophil numbers (**Supplementary Material, Figures S2F–J**). Thus, Gal-1 seems to exert a sex-specific regulation of leukocyte numbers during the resolution of inflammation.

### Gal-1 Shifts the Cytokine Production of Resolution-Phase Macrophages to Promote Their Reprogramming

**Figure 3:**
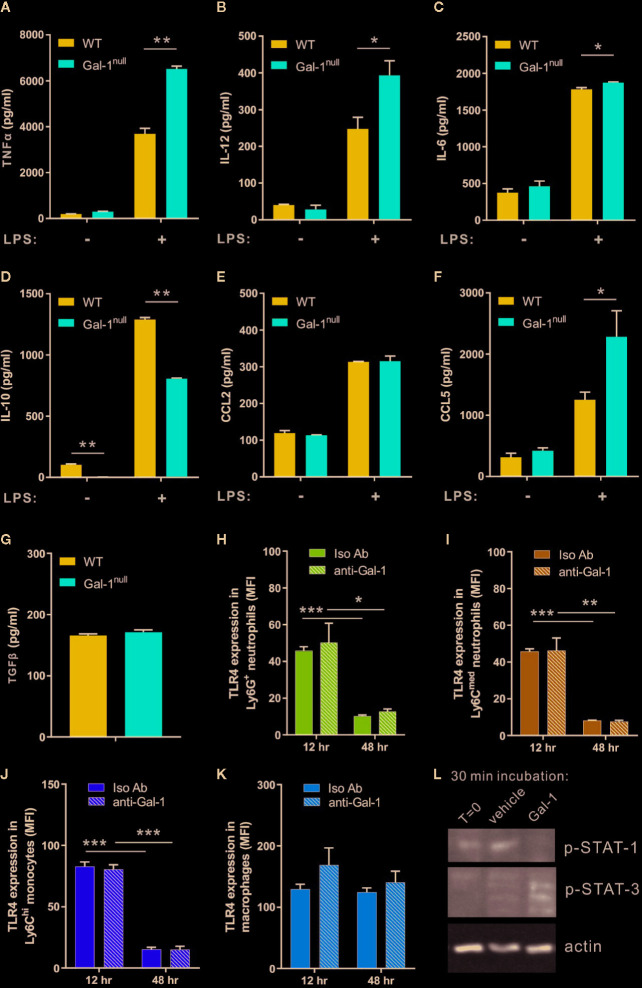
Gal-1 shifts the cytokine production of resolution-phase macrophages to promote their reprogramming. **(A-G)** Peritoneal macrophages were isolated from WT and Gal-1^null^ male mice 48 h PPI and then exposed to vehicle or LPS for 24 h. The concentrations of the following cytokines and chemokines were measured in the culture media by ELISA, n = 4–6: **(A)** TNF-α, **(B)** IL-12, **(C)** IL-6, **(D)** IL-10, **(E)** CCL2, and **(F)** CCL5. **(G)** TGF-β concentrations in the peritoneal fluids at 48 h were directly measured. **(H–K)** Peritoneal cells were collected from mice treated with anti-Gal-1 or Iso Ab immunostained as in **Figure 2**, as well as for TLR4, and analyzed by flow cytometry. Then, TLR4 expression on various leukocyte subsets at 12 and 48 h was determined. **(L)** Macrophages were isolated from peritoneal exudates of four mice at 48 h PPI and treated with vehicle or Gal-1 (4 μg/ml) for 30 min. Then, macrophages were lysed and their protein content was run by SDS-PAGE and blotted for phospho-STAT1, phospho-STAT3, and actin. Representative images are shown. Full blot images are shown in **Figure S8**. Altogether, the data represent at least two independent experiments and are presented as mean ± SEM. Statistical significance relative to WT mice was analyzed by two-tailed Student's *t*-test; *p < 0.05, **p < 0.01, ***p < 0.001.

The uptake of apoptotic cells by resolution phase macrophages results in a shift from pro-inflammatory to anti-inflammatory and pro-resolving cytokine secretion upon exposure to bacterial moieties (Voll et al., 1997; Fadok et al., 1998; Schif-Zuck et al., 2011; Kumaran Satyanarayanan et al., 2019). Previous studies indicate Gal-1 has immune-modulatory properties on macrophage cytokine secretion (Rostoker et al., 2013; Sundblad et al., 2017). Hence, we reasoned that Gal-1 deficiency in mice would lead to a delayed resolution of inflammation, culminating in hampered reprogramming of resolution-phase macrophages. To determine whether this is indeed the case, we isolated macrophages from zymosan A-challenged WT and Gal-1^null^ mice, and stimulated them with LPS *ex vivo* to determine their cytokine secretion. Our results indicate that non-stimulated macrophages secreted low levels of cytokines that mostly did not differ between the macrophage genotypes (**Figures 3A–F** and **Supplementary Material, Figures S3A–F**). Yet, we detected a reduced IL-10 secretion from non-stimulated male Gal-1^null^ macrophages (**Figure 3D**) in comparison to their WT counterparts. LPS stimulation, as a mimicry for bacterial exposure, led to a significant increase in cytokine secretion regardless of the macrophage genotype (**Figures 3A–F** and **Supplementary Material, Figures S3A–F**). In response to LPS stimulation, however, significant differences between Gal-1^null^ macrophages and WT macrophages were observed. LPS-stimulated macrophages from Gal-1^null^ males secreted higher levels of pro-inflammatory cytokines, such as TNF-α, IL-12, and IL-6, alongside lower levels of the anti-inflammatory cytokine IL-10 (**Figures 3A–D**). Inflammatory chemokines, which attract leukocytes to inflamed sites, are also modulated during macrophage reprogramming (Aswad et al., 2017). Our results revealed that LPS-stimulated macrophages from Gal-1^null^ males secreted similar levels of CCL2 and higher levels of CCL5 in comparison to their WT counterparts (**Figures 3E, F**). Moreover, we detected similar levels of TGF-β, which is an essential mediator in the resolution of inflammation (Bannenberg et al., 2005; Ariel and Timor, 2013), in the peritoneum of WT and Gal-1^null^ males (**Figure 3G**). These results did not support a complete debilitation of macrophage reprogramming in Gal-1^null^ mice, but rather an impaired cytokine-based pro-resolutive switch.

Modulation of macrophage reprogramming could take place through modulation of their TLR expression or changes in their intracellular signaling. To determine whether the hampered reprogramming of Gal-1^null^ resolution-phase macrophages is due to altered expression of TLR4, we determined TLR4 expression on various leukocyte subsets at 12 and 48 h PPI following neutralization of Gal-1. Our results (**Figures 3H–K**) show neither macrophages, monocytes or neutrophils modulate their TLR4 expression upon Gal-1 neutralization. However, treatment of resolution phase macrophages with recombinant Gal-1 resulted in a significant increase in STAT3 and a reduction in STAT1 activation (**Figure 3L**).

**Figure 4:**
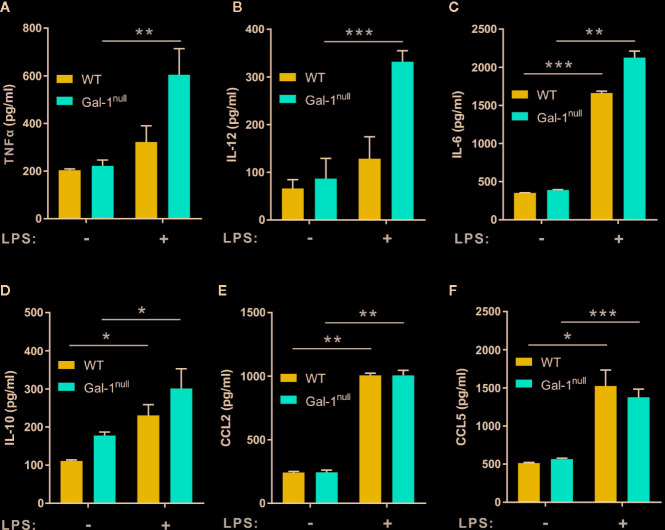
Gal-1^null^ naïve macrophages are more sensitive to LPS stimulation. Peritoneal naïve macrophages were isolated from unchallenged WT and Gal-1^null^ male mice and then exposed to vehicle or LPS for 24 h. The concentrations of the following cytokines and chemokines were measured in the culture media by ELISA: **(A)** TNF-α, **(B)** IL-12, **(C)** IL-6, **(D)** IL-10, **(E)** CCL2, and **(F)** CCL5. Altogether, the data represent two to three independent experiments and are presented as mean ± SEM, n = 4–8. Statistical significance relative to non-stimulated macrophages was analyzed by two-tailed Student's *t*-test; *p < 0.05, **p < 0.01, ***p < 0.001.

To determine whether Gal-1^null^ mice are hampered in macrophage reprogramming regardless of inflammation, we isolated naïve peritoneal macrophages from unchallenged WT and Gal-1^null^ mice and stimulated them with LPS *ex vivo*. Our results (**Figures 4A–F**) indicate that resident peritoneal macrophages from Gal-1^null^ mice secreted similar levels of TNF-α, IL-12, IL-6, CCL2, and CCL5 in comparison to their WT counterparts. Moreover, unlike resolution-phase Gal-1^null^ macrophages, their resident peritoneal counterparts did not show a reduction in IL-10 levels upon LPS exposure (**Figures 3D** and **4D**). Altogether, our results suggest that male Gal-1 is key for macrophage reprogramming exclusively during the resolution of inflammation, probably by modulating STAT signaling.

When examining whether the impact of Gal-1 on macrophage reprogramming is sex-dependent we found reduced secretion of TNF-α and IL-6, but not other cytokines or chemokines, from non-stimulated female Gal-1^null^ macrophages compared to their WT counterparts (**Supplementary Material, Figures S3A–F**). LPS-stimulated macrophages from Gal-1^null^ females did not demonstrate a hyperinflammatory phenotype as observed in Gal-1^null^ males. Rather we observed reduced secretion of TNF-α, IL-12, and IL-10, and similar levels of IL-6 in comparison to LPS-stimulated WT female macrophages (**Supplementary Material, Figures S3A–D**). LPS-stimulated macrophages from Gal-1^null^ females secreted similar levels of both CCL2 and CCL5 (**Supplementary Material, Figures S3E, F**) in comparison to their WT counterparts. Unexpectedly, Gal-1^null^ females demonstrated an increase in peritoneal TGF-β levels (**Supplementary Material, Figure S3G**) when compared to WT controls. The response of female resident macrophages to LPS was very similar to the response in males, albeit with significant increases in IL-10 and CCL5 secretion (**Supplementary Material, Figures S4A–F**). Thus, both resolution phase and resident macrophages from Gal-1^null^ females seem to be hyper responsive to LPS, rather than exert hampered reprogramming,

### Gal-1^null^ Mice Express Diminished Pro-Resolving Properties

**Figure 5:**
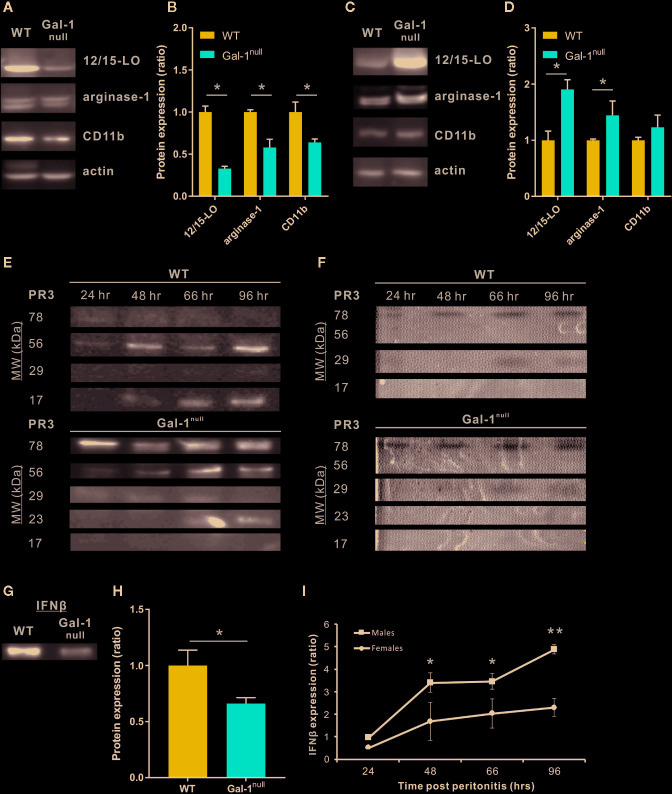
Gal-1^null^ mice express diminished pro-resolving properties. **(A, B)** Peritoneal macrophages from WT and Gal-1^null^ male mice were isolated at 48 h **(A, B)** and 66 h **(C, D)** PPI. The lysed macrophages were run by SDS-PAGE, followed by immunoblotting for 12/15-LO, arginase-1, CD11b, and actin as a loading control. **(A, C)** representative blot images. **(B, D)** graphs averaging protein expression after quantification by densitometry, normalizing to actin, and calculation of ratio to expression in WT macrophages. Full blot images are shown in **Figures S9** and **10**. **(E, F)** Peritoneal exudates were collected at 24, 48, 66, and 96 h PPI. **(E)** Equal volumes of cell-free fluids were run by SDS-PAGE, followed by immunoblotting for PR3; representative blot images. **(F)** Proteolytic activity of PR3 in exudates was examined by casein zymography; representative gel images. Full blot and zymography images are shown in **Figure S11**. **(G, H)** Equal volumes of cell-free fluids recovered at 48 h PPI were run by SDS-PAGE, followed by immunoblotting for IFN-β; representative blots **(G)** and mean ± SEM (H) Full blot images are shown in **Figure S12**. **(I)** Peritoneal fluids were obtained from WT and Gal-1^null^ male and female mice at 48 h PPI. IFN-β expression by Western blotting. Altogether, the data represent two to three independent experiments and are presented as mean ± SEM, n = 3–4. Statistical significance relative to WT mice **(B, D, H)** or between males and females **(I)** was analyzed by two-tailed Student's *t*-test; *p < 0.05, **p < 0.01.

During the course of peritonitis, monocytes/macrophages convert from an inflammatory CD11b^med^/arginase-1^low^/12/15-LO^neg^ phenotype to a reparative CD11b^high^/arginase-1^high^/12/15-LO^low^ phenotype, and then to a pro-resolving CD11b^low^/arginase-1^low^/12/15-LO^high^ phenotype (Bannenberg et al., 2005; Schif-Zuck et al., 2011; Ariel and Serhan, 2012; Kumaran Satyanarayanan et al., 2019). Hyperinflammation is often associated with loss of the pro-resolving, CD11b^low^ phenotype in macrophages (Ariel and Timor, 2013; Aswad et al., 2017; Kumaran Satyanarayanan et al., 2019). Moreover, we previously reported that Gal-1 administration during peritonitis promotes this pro-resolving, CD11b^low^ phenotype in macrophages and the production of the 12/15-LO product RvD1 (Rostoker et al., 2013). In line with our observation of defective resolution in Gal-1^null^ mice, our results revealed a lower expression of 12/15-LO, arginase-1, and CD11b in peritoneal macrophages 48 h PPI in males as compared with their WT counterparts (**Figures 5A, B**). Strikingly, at 66 h PPI we detected an increased expression of 12/15-LO and arginase-1 in male Gal-1^null^ macrophages compared with their WT counterparts (**Figures 5C, D**), suggesting a delay in macrophage conversion to the reparative and pro-resolving phenotypes during the resolution of inflammation. Notably, resolution-phase macrophages from female Gal-1^null^ mice only showed reduced 12/15-LO expression, but not in the other functional markers, and this reduction was limited to 48 h PPI, (**Supplementary Material, Figures S5A–D**).

High expression of the serine protease PR3 is an attribute of a neutrophilic inflammation that fails to resolve (Millet et al., 2015; Thieblemont et al., 2018). Therefore, we examined whether PR3 expression in peritoneal exudates is modulated by Gal-1. Our results indicate that Gal-1^null^ mice demonstrated a significant increase in various isoforms of PR3 in comparison to their WT counterparts (**Figure 5E** and **Supplementary Material, Figure S5E**). In addition to the well-known monomeric (29 kDa) and dimeric (56 kDa) PR3, additional forms of this enzyme appeared (78 and 23 kDa) and one form disappeared (17 kDa, probably a degradation product) (**Figure 5E** and **Supplementary Material, Figure S5E**). In support, zymography-based evaluation of PR3 proteolytic activity demonstrated a significant increase at 96 h PPI in Gal-1^null^ mice (**Figure 5F**, **Supplementary Material, Figures S5F** and **S6A, B**), suggesting that resolution of inflammation is blocked or delayed. In general, PR3 expression and proteolytic activity were stronger in females than males, regardless of genotype.

We recently reported that macrophage-derived IFN-β serves as a new effector cytokine in the resolution of inflammation (Kumaran Satyanarayanan et al., 2019). The IFN-β-IL-10 axis balances the activation of the STAT transcription factors to modulate cytokine production in macrophages (Chang et al., 2007; Hu et al., 2008) and our results in **Figure 3** indicate STAT1/3 are modulated in macrophages treated by Gal-1. Therefore, we examined whether Gal-1 deficiency affects IFN-β expression by resolution phase macrophages. In accord with the results presented here, we detected reduced levels of the IFN-β protein in the peritoneal exudates of Gal-1^null^ mice at 48 h PPI compared with WT controls (**Figures 5G, H** and **Supplementary Material, Figures S5G, H**). Moreover, female mice had lower levels of IFN-β in peritoneal exudates during the resolution phase, suggesting a sex-dependent role for this cytokine in resolving inflammation (**Figure 5I**). Taken together, our results suggest Gal-1 is an important effector molecule in regulating macrophage reparative and pro-resolving properties in a sex-dependent manner in resolving inflammation.

### IFN-β Reduces Neutrophil and Increases Macrophage Distribution in Resolving Exudates

**Figure 6:**
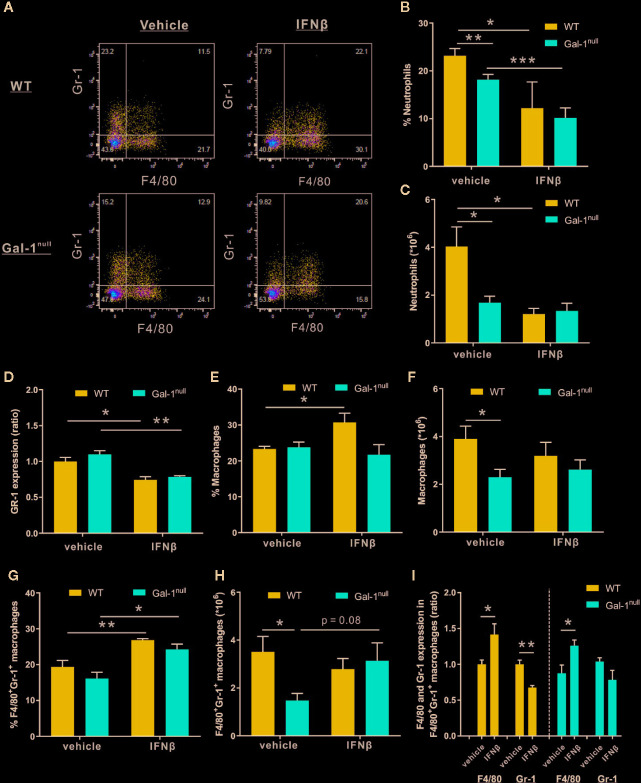
IFN-β reduces neutrophil and increases macrophage distribution in resolving exudates. WT and Gal-1^null^ male mice undergoing peritonitis were administered with IFN-β or vehicle 24 h PPI, and their peritoneal exudates were collected 24 h later. Peritoneal leukocytes thereof were enumerated, immunostained for Gr-1 and F4/80, and analyzed by flow cytometry. **(A)** Representative dot plots. **(B)** The percentage of neutrophils. **(C)** The total number of neutrophils **(D)** The ratio of Gr-1 expression, based on MFI. **(E)** The percentage of macrophages **(F)** The total number of macrophages. **(G)** The percentage of F4/80^+^Gr-1^+^ macrophages. **(H)** The total number of F4/80^+^Gr-1^+^ macrophages. **(I)** The ratio of F4/80 and Gr-1 expression, based on MFI, in F4/80^+^Gr-1^+^ macrophages. Data are presented as mean ± SEM, n = 4. Statistical significance relative to vehicle-treated mice was analyzed by two-tailed Student's *t*-test; *p < 0.05, **p < 0.01.

The reduction in IFN-β expression in Gal-1^null^ mice suggests that IFN-β acts downstream of Gal-1 in resolving acute inflammation. Due to the hampered resolution of inflammation in Gal-1^null^ males, we exclusively examined the rescue of resolution keys in these mice by exogenous IFN-β. To this end, we treated WT and Gal-1^null^ mice with IFN-β at 24 h PPI, followed by isolation of peritoneal leukocytes at 48 h PPI. Our results indicate that IFN-β treatment *in vivo* was not able to restore the decline in peritoneal neutrophil numbers in Gal-1^null^ mice (**Figures 6A–C**). Rather, it further reduced the percentage of neutrophils and the expression of the neutrophil marker Gr-1 in both WT and Gal-1^null^ mice (**Figures 6B, D**). These findings are in accord with the facilitation of neutrophil apoptosis and efferocytosis by macrophages by IFN-β (Kumaran Satyanarayanan et al., 2019). On the other hand, IFN-β treatment *in vivo* increased the percentage of macrophages in WT mice, but not in Gal-1^null^ mice (**Figure 6E**). This observation was not reflected in macrophage numbers (**Figure 6F**). Of interest, we detected an increase in the percentage of double-positive macrophages (F4/80^+^Gr-1^+^) following IFN-β pre-treatment *in vivo* in both WT and Gal-1^null^ mice (**Figure 6G**). This observation was not reflected in double-positive macrophage numbers (**Figure 6H**). Moreover, this subset of macrophages expressed elevated levels of the macrophage marker F4/80 in response to IFN-β, regardless of genotype (**Figure 6I**). In WT mice, but not in Gal-1^null^ mice, this subset expressed lower levels of neutrophil marker Gr-1 (**Figure 6I**). IFN-β treatment *in vivo* did not affect the percentage of peritoneal eosinophils in the exudates of Gal-1^null^ mice (data not shown). In summary, IFN-β treatment *in vivo* did not normalize the peritoneal leukocyte distribution of Gal-1^null^ mice, yet, it increased the numbers of double-positive macrophages.

### IFN-β Rescues Hampered Keys of Resolution of Inflammation in Gal-1^null^ Mice

**Figure 7:**
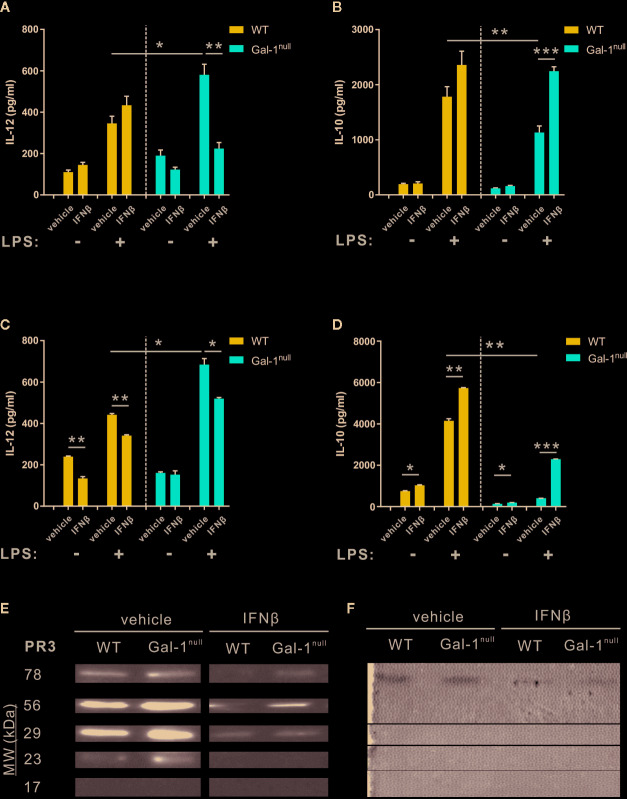
IFN-β rescues hampered keys of resolution of inflammation in Gal-1^null^ mice. **(A, B)** WT and Gal-1^null^ male mice undergoing peritonitis were administered with IFN-β or vehicle 24 h PPI, and their peritoneal exudates were collected 24 h later. Macrophages were isolated from the exudates and exposed to vehicle or LPS for 24 h. **(C, D)** Peritoneal macrophages were isolated from WT and Gal-1^null^ male mice 66 h PPI. Then, the macrophages were treated with IFN-β or vehicle, and in parallel exposed to vehicle or LPS for 24 h. The concentrations of the following cytokines and chemokines were measured in the culture media by ELISA: **(A, C)** IL-12, and **(B, D)** IL-10. **(E, F)** Mice were administered with IFN-β or a vehicle 24 h PPI, and peritoneal exudates were collected 48 h PPI. **(E)** Equal volumes of cell-free fluids were run by SDS-PAGE, followed by immunoblotting for PR3; representative blot images. **(F)** Proteolytic activity of PR3 in exudates was examined by casein zymography; representative gel images are shown. Full blot and zymography images are shown in **Figure S13**. Data are representative of two to three independent experiments and are presented as mean ± SEM, n = 4–8. Statistical significance relative to vehicle-treated mice or between LPS-stimulated WT and Gal-1^null^ macrophages was analyzed by two-tailed Student's *t*-test; *p < 0.05, **p < 0.01, ***p < 0.001.

Next, we sought to determine whether exogenous IFN-β can rescue the hampered resolution keys observed in Gal-1^null^ mice. To this end, mice undergoing peritonitis received IFN-β at 24 h PPI, and at 48 h PPI peritoneal macrophages were isolated and stimulated with LPS. In accord with previous experiments, LPS-stimulated Gal-1^null^ macrophages secreted higher IL-12 levels and lower IL-10 levels compared with WT macrophages (**Figures 7A, B**). IFN-β treatment *in vivo* did not significantly affect cytokine secretion from LPS-stimulated WT macrophages (**Figure 7**). However, it significantly reduced IL-12 secretion and increased IL-10 secretion from LPS-stimulated Gal-1^null^ macrophages (**Figures 7A, B**). Thus, IFN-β restored the hampered macrophage reprogramming index (Aswad et al., 2017) of Gal-1^null^ mice. Unexpectedly, IFN-β treatment *in vivo* did not affect TNF-α and IL-6 secretion from LPS-stimulated macrophages (data not shown). To determine whether IFN-β directly affects Gal-1^null^ macrophages, we treated peritoneal macrophages, isolated at 66 h PPI, *with* IFN-β *ex vivo*. As expected, LPS-stimulated Gal-1^null^ macrophages secreted higher IL-12 levels and lower IL-10 levels in comparison with their WT counterparts (**Figures 3B, D** and **Figures 7C, D**). Strikingly, IFN-β treatment *ex vivo* also rescued the reprogramming index of LPS-stimulated Gal-1^null^ macrophages, by reducing IL-12 levels and increasing IL-10 levels (**Figures 7C, D**). Notably, IFN-β reduced IL-12 secretion in WT macrophages and increased IL-10 secretion in WT and Gal-1^null^ macrophages also in the absence of LPS stimulation (**Figures 7C, D**). Thus, IFN-β rescues hampered reprogramming in Gal-1^null^ resolution-phase macrophages.

8Next, we determined whether IFN-β rescues the increased PR3 expression and proteolytic activity in the peritoneal exudates of male Gal-1^null^ mice as compared with male WT controls. In line with our previous results (**Figure 5C**), vehicle-treated Gal-1^null^ males expressed higher levels of PR3 compared with their WT counterparts (**Figure 7E**). IFN-β treatment *in vivo* significantly reduced PR3 expression in both Gal-1^null^ and WT mice. This response was particularly impactful in Gal-1^null^ mice, where the 23 kDa form of PR3 completely disappeared (**Figure 7E**). Along these lines, IFN-β treatment *in vivo* significantly inhibited the proteolytic activity of PR3 measured by zymography analysis (**Figure 7F** and **Supplementary Material, Figure S6C**). Altogether, our results suggest IFN-β rescues hampered resolution of inflammation in Gal-1^null^ mice.

## Discussion

Accumulating evidence from various experimental studies points at the lectin Gal-1 as a central immunomodulator in various inflammatory and autoimmune disorders (Rabinovich and Croci, 2012; Sundblad et al., 2017). By inhibiting leukocyte motility and activation (Norling et al., 2008; Cooper et al., 2010), Gal-1 regulates different types of immune responses, from immune-tolerance, exploited by cancer cells, to acute and chronic inflammation (Sundblad et al., 2017; Chou et al., 2018). Gal-1 also elicits type II anti-inflammatory responses in macrophages and microglia (Correa et al., 2003; Starossom et al., 2012; Abebayehu et al., 2017), therefore mediating pro-resolving and neuroprotective effects (Kurihara et al., 2010; Rostoker et al., 2013). In this study, we detected an elevation of Gal-1 expression in F4/80^+^Ly6C^-^ peritoneal macrophages accompanied with decreased expression in their monocyte precursors 48 h after the onset of spontaneously-resolving peritonitis (Bannenberg et al., 2005; Cash et al., 2009), indicating a possible role in the initiation of the resolution of inflammation. Of interest, peritoneal resident macrophages expressed relatively low levels of Gal-1, but neutralization of this protein from other sources lead to a significant reduction in the numbers of Tim4^+^ resident peritoneal macrophages, possibly by diminishing their proliferation or survival. Indeed, we and others, have shown that Gal-1 enhances the resolution of inflammation (Rostoker et al., 2013; Seropian et al., 2013; Sundblad et al., 2018; Law et al., 2020). Thus, we reasoned that peritonitis-inflicted Gal-1^null^ mice would display hampered resolution, culminating in hyperinflammation and reduced macrophage reprogramming. Our analysis is divided to two parts. In the first part, in **Figure 2** and **Supplementary Material, Figure S2**, we distinguished immature monocytes (Ly6C^+^F4/80^lo^) from mature macrophages (Ly6C^-^F4/80^+^) and found a reduction in mature macrophage numbers and F4/80 expression in Gal-1^null^ mice. However, the observed difference in macrophages differentiation from inflammatory monocytes can only partly explain the differences in macrophage cytokine production we found in Gal-1^null^ macrophages, and this is probably not due to shifts from M1 to M2. It is more likely related to the changes in the expression of arginase 1 and 12/15-LO that were detected in the second part of our analysis (**Figure 5** and **Supplementary Material, Figure S5**). Overall, our findings supported the hypothesis that Gal-1^null^ resolution phase macrophages are hyperinflammatory with imbalanced reprogramming since they secreted higher levels of pro-inflammatory cytokines, such as TNF-α, IL-6, and IL-12, while secreting lower levels of the anti-inflammatory cytokine IL-10 upon LPS exposure. This phenomenon was not associated with any changes in TLR4 expression, but Gal-1 treatment *ex vivo* induced a reduction in STAT1 activation while STAT3 was activated. Thus, Gal-1^null^ macrophages had a reduced reprogramming index than their WT counterparts with possible contribution from modulated STAT signaling.

Along the lines of hampered reprogramming, Gal-1^null^ macrophages had a delayed expression of the pro-resolving macrophage-expressed enzyme 12/15-LO, suggesting an aberrant production of resolution-promoting SPM (Rostoker et al., 2013; Ackermann et al., 2017), and the anti-inflammatory/reparative macrophage enzyme arginase-1. Although pro-resolving macrophages normally do not express arginase-1, type II macrophages express it as a bypass for NO production that characterizes type I macrophages (Freire-De-Lima et al., 2006). Arginase-1 is expressed by CD11b^high^ macrophages during the early phase of resolution of peritonitis. Therefore, diminished expression of this enzyme, together with reduced CD11b, reflects dysregulated maturation of macrophages in this model (Schif-Zuck et al., 2011; Lumbroso et al., 2018). Since Gal-1 treatment *in vitro* rapidly enhances arginase-1 expression in macrophages (Rostoker et al., 2013), our results of lower arginase-1 expression at first, followed by higher expression later, are consistent with hampered macrophage maturation and reprogramming from inflammatory to anti-inflammatory/reparative phenotype in Gal-1^null^ mice.

Interestingly, the numbers of peritoneal leukocytes, including macrophages, neutrophils, and eosinophils, declined in peritonitis-inflicted Gal-1^null^ mice, whereas neutralization of extracellular Gal-1 resulted in reduced peritoneal neutrophil numbers during the resolution, but not the onset, of inflammation. Gal-1 neutralization also exclusively reduced its expression by resolution phase macrophages but did not affect their numbers at 48 h, possibly due to residual activity of inaccessible stores of this protein. It remains to be determined whether Gal-1^null^ mice have a lower capacity to produce leukocytes upon inflammatory stimuli, and/or whether they exert impaired leukocyte recruitment to inflammation sites. Accordingly, Gal-1 treatment *in vitro* enhances myeloid cell differentiation (Vas et al., 2005), to account for the lower leukocyte numbers in Gal-1^null^ mice. On the other hand, Gal-1 treatment was shown to inhibit or promote myeloid cell migration, depending on the experimental design (Malik et al., 2009; Paclik et al., 2011; Auvynet et al., 2013). Gal-1 administration slows down neutrophil recruitment *in vivo* (La et al., 2003; Gil et al., 2010), however, with time it increases monocyte/macrophage recruitment (Gil et al., 2010). Under non-inflammatory conditions Gal-1 functions as a chemoattractant by binding CD43 (Auvynet et al., 2013), and might act similarly during the resolution of peritonitis when chemoattractant levels are below detection (Bannenberg et al., 2005). Thus, temporally-changing regulation may also play a role in our inflammatory model. We focused on examining leukocyte populations and the cytokine repertoire at 48 h PPI, when Gal-1 expression is at its peak. Examining leukocyte populations and cytokine-secretion repertoire at other time points could provide valuable information on how the Gal-1 feedback loop regulates the inflammatory response in a timely manner.

PR3 plays pleiotropic roles in escalating inflammatory response (Kettritz, 2016; Thieblemont et al., 2018); therefore, we evaluated PR3 expression and proteolytic activity, and detected increased expression and activity in Gal-1^null^ mice. Of note, we detected increased expression of an additional ~80 kDa form of PR3 that seems to be released from late apoptotic PMN (data not shown) in Gal-1^null^ mice. Previous reports show that PR3 shapes the microenvironment (Millet et al., 2015), allowing the recruitment of inflammatory cells, which was not the case in our study. However, leukocyte migration and PR3 release usually precede the resolution phase and takes place at 4 to 12 h PPI. The increases in free PR3 levels in the peritoneum during the resolution phase seem to not be related to the number of peritoneal neutrophils that decrease in Gal-1^null^ mice. Rather, since IFN-β promotes neutrophil caspase-dependent apoptosis and efferocytosis (Kumaran Satyanarayanan et al., 2019; Law et al., 2020), its down-regulation in Gal-1^null^ mice could divert neutrophils to a necroptotic state that allows release of PR3 to the resolving tissue. PR3, in turn, limits complement-mediated efferocytosis (Tacnet-Delorme et al., 2018) and macrophage reprogramming that is observed in Gal-1/IFN-β-deficient macrophages as well (**Figure 3** and (Millet et al., 2015; Kumaran Satyanarayanan et al., 2019). Another plausible explanation for the increase in PR3 release in Gal-1^null^ mice could emanate from the role of PR3 in regulating caspase-dependent neutrophil apoptosis (Loison et al., 2014) that is promoted by Gal-1/ IFN-β. The reduced neutrophil apoptosis observed in Gal-1/IFN-β-deficient mice could be due to release of lysosomal PR3 to the extracellular milieu rather than to the neutrophil cytosol, and this miss-localization of PR3 would concomitantly diminish PMN apoptosis and efferocytosis, and macrophage reprogramming. Reduced IFN-β could also play a role in the decline in neutrophil and macrophage numbers in Gal-1^null^ mice. Since IFN-β was recently shown to be involved in the attraction of both neutrophils and macrophages to the peritoneum during zymosan A peritonitis (Kumaran Satyanarayanan et al., 2019), its down-regulation in Gal-1^null^ mice could lead to their diminished recruitment.

Notably, peritonitis-inflicted Gal-1^null^ females exhibited a different phenotype, which was generally less hyperinflammatory as compared with Gal-1^null^ males. Leukocyte distribution, except for eosinophils, was unaffected, and secretion of the pro-inflammatory cytokines TNF-α and IL-12 demonstrated a reduction rather than an increase. Unlike in males, TGF-β levels in the peritoneum, which are normally associated with inflammation resolution or tissue fibrosis (Bannenberg et al., 2005; Ariel and Timor, 2013), presented an increase. Additionally, apart from initial reduced expression of 12/15-LO, expression of other macrophage reprogramming markers was not affected in Gal-1^null^ females. Conversely, Gal-1^null^ macrophages from challenged females secreted lower levels of IL-10, and Gal-1^null^ macrophages from unchallenged females secreted higher levels of the pro-inflammatory cytokines TNF-α and IL-12. PR3 expression and proteolytic activity were substantially stronger in Gal-1^null^ females. Taken together, some features in Gal-1^null^ females were consistent with a hyperinflammatory phenotype and some did not. It has been shown that estrogen accelerates the resolution of inflammation (Villa et al., 2015); therefore, the consequence of Gal-1 deficiency might not be as detrimental in females as in males. Of note, the importance of Gal-1 in fetomaternal immunotolerance and the existence of estrogen response element on the promoter of the Gal-1 gene imply a complex sex-dependent immunoregulation of this lectin (Than et al., 2008; Sundblad et al., 2017).

Recently, we reported that IFN-β^null^ macrophages display hampered reprogramming, resulting in higher secretion of pro-inflammatory cytokines and lower levels of anti-inflammatory cytokines, whereas IFN-β treatment *in vivo* and *ex vivo* leads to the opposite response (Kumaran Satyanarayanan et al., 2019). Thus, IFN-β regulates macrophage reprogramming in a similar manner to Gal-1 (Rostoker et al., 2013). In support, IFN-β seems to be a downstream effector of Gal-1, based on our observation that IFN-β expression was downregulated in both Gal-1^null^ males and females. Furthermore, the findings of this study revealed that IFN-β treatment *in vivo* or *ex vivo* rescued in part the hampered resolution culminating from Gal-1 deficiency. IFN-β treatment reduced the LPS-stimulated secretion of pro-inflammatory IL-12, but not TNF-α and IL-6, and increased the secretion of anti-inflammatory IL-10 from Gal-1^null^ macrophages. In addition, it reduced PR3 expression and proteolytic activity in exudates from peritonitis-inflicted mice. The downstream signaling that links Gal-1 and IFN-β activities remains unknown; as far as we know, no data in the literature links between IFN-β and other galectins as well. Notwithstanding, the immunomodulatory effects of Gal-1 are mediated only in part by IFN-β, suggesting the existence of additional downstream effectors, and IFN-β by itself has effects that are Gal-1–independent. For example, IFN-β treatment led to a further decrease in neutrophil numbers and Gr-1 expression in Gal-1^null^ mice rather than to a rescue of the aberrant phenotype. This outcome is expected, considering that IFN-β promotes neutrophil apoptosis during cancer development as well as the resolution of inflammation (Andzinski et al., 2015; Kumaran Satyanarayanan et al., 2019). Of interest, IFN-β increased F4/80 expression and the relative proportion of macrophages in WT and Gal-1^null^ mice. This observation could be a result of enhanced macrophage recruitment and/or retention. Our previous report indicated a diminished efferocytosis in macrophages as well as a lower degree of neutrophil apoptosis in IFN-β^null^ mice (Kumaran Satyanarayanan et al., 2019). Because Gal-1^null^ macrophages had a lower expression of IFN-β, this pro-resolving cytokine could enhance their efferocytosis, together with the increased abundance of apoptotic neutrophils. IFN-β has also been shown to increase monocyte/macrophage migration and recruitment to inflammatory sites (Goossens et al., 2010; Venkatesh et al., 2013; Ruiz Silva et al., 2016). Whether IFN-β rescues macrophage recruitment and efferocytosis in Gal-1^null^ mice is a subject for future studies. Notably, regardless of genotype, IFN-β increased the fraction of double-positive F4/80^+^Gr-1^+^ macrophages. Although the nature of this sub-population under the experimental design of our study is undeciphered, GR-1^+^ macrophages are phagocytic cells that can efferocytose apoptotic neutrophils, thereby taking an active part in the resolution of inflammation (Mordue and Sibley, 2003; Kataru et al., 2009).

**Figure 8:**
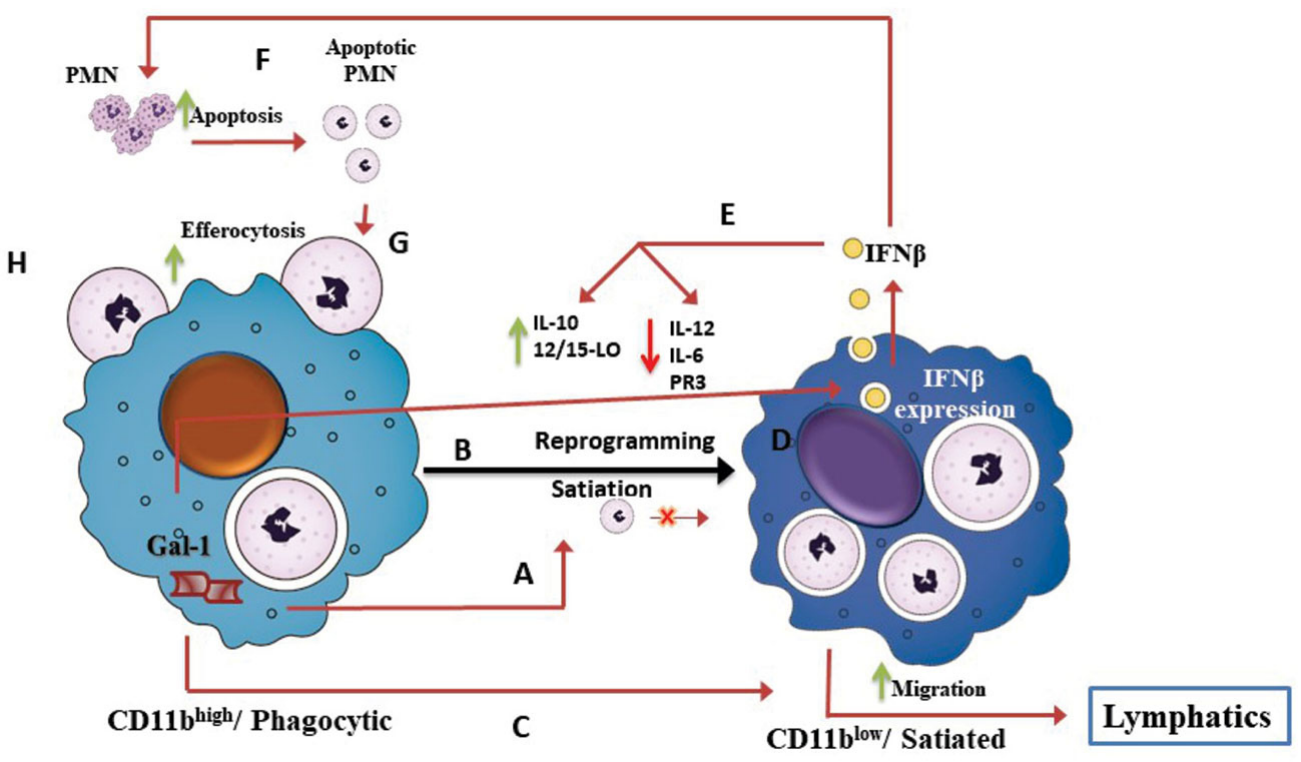
The Gal-1-IFN-β axis drives macrophage reprogramming during the resolution of inflammation. Schematic illustration of our current understanding of the role of the Gal-1-IFN-β axis in macrophage reprogramming during resolution of inflammation, based on our current findings and recent reports (Schif-Zuck et al., 2011; Ariel and Timor, 2013; Rostoker et al., 2013; Kumaran Satyanarayanan et al., 2019). Acute inflammation is characterized by recruitment of short-lived polymorphonuclear (PMN) cells, which in turn, undergo apoptosis and engulfment by pro-inflammatory monocytes/macrophages that converts them to reparative/phagocytic/CD11b^high^ macrophages. CD11b^high^/phagocytic macrophages produce Gal-1 **(A)** that promotes macrophage satiation and reprogramming to the pro-resolving/CD11b^low^ phenotype **(B)**. Gal-1 also promotes macrophage migration to lymphatics **(C)**. Our study suggests that Gal-1-induced apoptotic PMN uptake triggers a positive feedback loop, mediated by IFN-β. The uptake of apoptotic PMN triggers IFN-β production and secretion **(D)**. IFN-β, in turn, mediates Gal-1-induced macrophage reprogramming **(E)**, PMN apoptosis **(F)**, and efferocytosis **(G)**. Validation that all these steps of the Gal-1-IFN-β feedback loop take place in a coordinated fashion requires further investigation.

In summary, our study revealed a novel Gal-1-IFN-β axis in mouse macrophages that plays an immunomodulatory role during the resolution of inflammation, driving macrophage reprogramming (**Figure 8**). These results have therapeutic implications, since both Gal-1 and IFN-β can exert pro-resolutive actions in various types of chronic inflammatory diseases.

## Data Availability Statement

The datasets generated for this study are available on request to the corresponding author.

## Ethics Statement

The animal study was reviewed and approved by The Ethics committee for animal experimentation, University of Haifa, Haifa, Israel.

## Author Contributions

HY, IP-Z, and SB isolated macrophages and exudates from murine peritonitis, performed activation and ELISA for cytokines, Western blotting for phenotypic markers, flow cytometry, and zymography. SS-Z assisted in various aspects of the experimental design and experimentation. JP-S and GR provided reagents and assisted in the design of the study. AA designed the study, assisted in analyzing the data and wrote the manuscript.

## Funding

The study was supported by grants from the Israel Science Foundation (Grant No. 678/13), the Rosetrees Trust and the Wolfson Family Charitable Trust.

## Conflict of Interest

The authors declare that the research was conducted in the absence of any commercial or financial relationships that could be construed as a potential conflict of interest.

## References

[B1] Abebayehu D. Spence A. Boyan B. D. Schwartz Z. Ryan J. J. Mcclure M. J. (2017). Galectin-1 promotes an M2 macrophage response to polydioxanone scaffolds. J. BioMed. Mater. Res. A 105, 2562–2571. 10.1002/jbm.a.36113 28544348 PMC5563977

[B2] Ackermann J. A. Hofheinz K. Zaiss M. M. Kronke G. (2017). The double-edged role of 12/15-lipoxygenase during inflammation and immunity. Biochim. Biophys. Acta Mol. Cell Biol. Lipids 1862, 371–381. 10.1016/j.bbalip.2016.07.014 27480217

[B3] Andzinski L. Wu C. F. Lienenklaus S. Kroger A. Weiss S. Jablonska J. (2015). Delayed apoptosis of tumor associated neutrophils in the absence of endogenous IFN-beta. Int. J. Cancer 136, 572–583. 10.1002/ijc.28957 24806531

[B4] Ariel A. Serhan C. N. (2012). New Lives Given by Cell Death: Macrophage Differentiation Following Their Encounter with Apoptotic Leukocytes during the Resolution of Inflammation. Front. Immunol. 3, 4. 10.3389/fimmu.2012.00004 22566890 PMC3342362

[B5] Ariel A. Timor O. (2013). Hanging in the balance: endogenous anti-inflammatory mechanisms in tissue repair and fibrosis. J. Pathol. 229, 250–263. 10.1002/path.4108 23007838

[B6] Arthur C. M. Baruffi M. D. Cummings R. D. Stowell S. R. (2015). Evolving mechanistic insights into galectin functions. Methods Mol. Biol. 1207, 1–35. 10.1007/978-1-4939-1396-1_1 25253130 PMC5755386

[B7] Aswad M. Assi S. Schif-Zuck S. Ariel A. (2017). CCL5 Promotes Resolution-Phase Macrophage Reprogramming in Concert with the Atypical Chemokine Receptor D6 and Apoptotic Polymorphonuclear Cells. J. Immunol. 199, 1393–1404. 10.4049/jimmunol.1502542 28674178

[B8] Atri C. Guerfali F. Z. Laouini D. (2018). Role of Human Macrophage Polarization in Inflammation during Infectious Diseases. Int. J. Mol. Sci. 19, 1801–1816. 10.3390/ijms19061801 PMC6032107 29921749

[B9] Auvynet C. Moreno S. Melchy E. Coronado-Martinez I. Montiel J. L. Aguilar-Delfin I. (2013). Galectin-1 promotes human neutrophil migration. Glycobiology 23, 32–42. 10.1093/glycob/cws128 22942212

[B10] Bannenberg G. L. Chiang N. Ariel A. Arita M. Tjonahen E. Gotlinger K. H. (2005). Molecular circuits of resolution: formation and actions of resolvins and protectins. J. Immunol. 174, 4345–4355. 10.4049/jimmunol.174.7.4345 15778399

[B11] Bratton D. L. Henson P. M. (2011). Neutrophil clearance: when the party is over, clean-up begins. Trends Immunol. 32, 350–357. 10.1016/j.it.2011.04.009 21782511 PMC3151332

[B12] Butenko S. Satyanarayanan S. K. Assi S. Schif-Zuck S. Sher N. Ariel A. (2020). Transcriptomic Analysis of Monocyte-Derived Non-Phagocytic Macrophages Favors a Role in Limiting Tissue Repair and Fibrosis. Front. Immunol. 11, 405. 10.3389/fimmu.2020.00405 32296415 PMC7136412

[B13] Cash J. L. White G. E. Greaves D. R. (2009). Chapter 17. Zymosan-induced peritonitis as a simple experimental system for the study of inflammation. Methods Enzymol. 461, 379–396. 10.1016/S0076-6879(09)05417-2 19480928

[B14] Chang E. Y. Guo B. Doyle S. E. Cheng G. (2007). Cutting edge: involvement of the type I IFN production and signaling pathway in lipopolysaccharide-induced IL-10 production. J. Immunol. 178, 6705–6709. 10.4049/jimmunol.178.11.6705 17513714

[B15] Chou F. C. Chen H. Y. Kuo C. C. Sytwu H. K. (2018). Role of Galectins in Tumors and in Clinical Immunotherapy. Int. J. Mol. Sci. 19, 430–463. 10.3390/ijms19020430 PMC5855652 29389859

[B16] Cooper D. Ilarregui J. M. Pesoa S. A. Croci D. O. Perretti M. Rabinovich G. A. (2010). Multiple functional targets of the immunoregulatory activity of galectin-1: Control of immune cell trafficking, dendritic cell physiology, and T-cell fate. Methods Enzymol. 480, 199–244. 10.1016/S0076-6879(10)80011-4 20816212

[B17] Correa S. G. Sotomayor C. E. Aoki M. P. Maldonado C. A. Rabinovich G. A. (2003). Opposite effects of galectin-1 on alternative metabolic pathways of L-arginine in resident, inflammatory, and activated macrophages. Glycobiology 13, 119–128. 10.1093/glycob/cwg010 12626408

[B18] Dalli J. Serhan C. N. (2017). Pro-Resolving Mediators in Regulating and Conferring Macrophage Function. Front. Immunol. 8, 1400. 10.3389/fimmu.2017.01400 29163481 PMC5671941

[B19] Elliott M. R. Koster K. M. Murphy P. S. (2017). Efferocytosis Signaling in the Regulation of Macrophage Inflammatory Responses. J. Immunol. 198, 1387–1394. 10.4049/jimmunol.1601520 28167649 PMC5301545

[B20] Fadok V. A. Bratton D. L. Konowal A. Freed P. W. Westcott J. Y. Henson P. M. (1998). Macrophages that have ingested apoptotic cells in vitro inhibit proinflammatory cytokine production through autocrine/paracrine mechanisms involving TGF-beta, PGE2, and PAF. J. Clin. Invest. 101, 890–898. 10.1172/JCI1112 9466984 PMC508637

[B21] Filep J. G. (2013). Resolution of inflammation: leukocytes and molecular pathways as potential therapeutic targets. Front. Immunol. 4, 256. 10.3389/fimmu.2013.00256 23986764 PMC3753553

[B22] Freire-De-Lima C. G. Xiao Y. Q. Gardai S. J. Bratton D. L. Schiemann W. P. Henson P. M. (2006). Apoptotic cells, through transforming growth factor-beta, coordinately induce anti-inflammatory and suppress pro-inflammatory eicosanoid and NO synthesis in murine macrophages. J. Biol. Chem. 281, 38376–38384. 10.1074/jbc.M605146200 17056601

[B23] Gieseck R. L. 3rd Wilson M. S. Wynn T. A. (2018). Type 2 immunity in tissue repair and fibrosis. Nat. Rev. Immunol. 18, 62–76. 10.1038/nri.2017.90 28853443

[B24] Gil C. D. Gullo C. E. Oliani S. M. (2010). Effect of exogenous galectin-1 on leukocyte migration: modulation of cytokine levels and adhesion molecules. Int. J. Clin. Exp. Pathol. 4, 74–84. 21228929 PMC3016105

[B25] Goossens P. Gijbels M. J. Zernecke A. Eijgelaar W. Vergouwe M. N. Van Der Made I. (2010). Myeloid type I interferon signaling promotes atherosclerosis by stimulating macrophage recruitment to lesions. Cell Metab. 12, 142–153. 10.1016/j.cmet.2010.06.008 20674859

[B26] Greenlee-Wacker M. C. (2016). Clearance of apoptotic neutrophils and resolution of inflammation. Immunol. Rev. 273, 357–370. 10.1111/imr.12453 27558346 PMC5000862

[B27] Headland S. E. Norling L. V. (2015). The resolution of inflammation: Principles and challenges. Semin. Immunol. 27, 149–160. 10.1016/j.smim.2015.03.014 25911383

[B28] Hu X. Chakravarty S. D. Ivashkiv L. B. (2008). Regulation of interferon and Toll-like receptor signaling during macrophage activation by opposing feedforward and feedback inhibition mechanisms. Immunol. Rev. 226, 41–56. 10.1111/j.1600-065X.2008.00707.x 19161415 PMC2630590

[B29] Isobe Y. Kato T. Arita M. (2012). Emerging roles of eosinophils and eosinophil-derived lipid mediators in the resolution of inflammation. Front. Immunol. 3, 270. 10.3389/fimmu.2012.00270 22973272 PMC3428698

[B30] Kataru R. P. Jung K. Jang C. Yang H. Schwendener R. A. Baik J. E. (2009). Critical role of CD11b+ macrophages and VEGF in inflammatory lymphangiogenesis, antigen clearance, and inflammation resolution. Blood 113, 5650–5659. 10.1182/blood-2008-09-176776 19346498

[B31] Kettritz R. (2016). Neutral serine proteases of neutrophils. Immunol. Rev. 273, 232–248. 10.1111/imr.12441 27558338

[B32] Krzyszczyk P. Schloss R. Palmer A. Berthiaume F. (2018). The Role of Macrophages in Acute and Chronic Wound Healing and Interventions to Promote Pro-wound Healing Phenotypes. Front. Physiol. 9, 419. 10.3389/fphys.2018.00419 29765329 PMC5938667

[B33] Kumaran Satyanarayanan S. El Kebir D. Soboh S. Butenko S. Sekheri M. Saadi J. (2019). IFN-beta is a macrophage-derived effector cytokine facilitating the resolution of bacterial inflammation. Nat. Commun. 10, 3471. 10.1038/s41467-019-10903-9 31375662 PMC6677895

[B34] Kurihara D. Ueno M. Tanaka T. Yamashita T. (2010). Expression of galectin-1 in immune cells and glial cells after spinal cord injury. Neurosci. Res. 66, 265–270. 10.1016/j.neures.2009.11.008 19941913

[B35] La M. Cao T. V. Cerchiaro G. Chilton K. Hirabayashi J. Kasai K. (2003). A novel biological activity for galectin-1: inhibition of leukocyte-endothelial cell interactions in experimental inflammation. Am. J. Pathol. 163, 1505–1515. 10.1016/S0002-9440(10)63507-9 14507657 PMC1868297

[B36] Law H. L. Wright R. D. Iqbal A. J. Norling L. V. Cooper D. (2020). A Pro-resolving Role for Galectin-1 in Acute Inflammation. Front. Pharmacol. 11, 274. 10.3389/fphar.2020.00274 32265698 PMC7098973

[B37] Loison F. Zhu H. Karatepe K. Kasorn A. Liu P. Ye K. (2014). Proteinase 3-dependent caspase-3 cleavage modulates neutrophil death and inflammation. J. Clin. Invest. 124, 4445–4458. 10.1172/JCI76246 25180606 PMC4191030

[B38] Lumbroso D. Soboh S. Maimon A. Schif-Zuck S. Ariel A. Burstyn-Cohen T. (2018). Macrophage-Derived Protein S Facilitates Apoptotic Polymorphonuclear Cell Clearance by Resolution Phase Macrophages and Supports Their Reprogramming. Front. Immunol. 9, 358. 10.3389/fimmu.2018.00358 29545796 PMC5837975

[B39] Malik R. K. Ghurye R. R. Lawrence-Watt D. J. Stewart H. J. (2009). Galectin-1 stimulates monocyte chemotaxis via the p44/42 MAP kinase pathway and a pertussis toxin-sensitive pathway. Glycobiology 19, 1402–1407. 10.1093/glycob/cwp077 19561030

[B40] Millet A. Martin K. R. Bonnefoy F. Saas P. Mocek J. Alkan M. (2015). Proteinase 3 on apoptotic cells disrupts immune silencing in autoimmune vasculitis. J. Clin. Invest. 125, 4107–4121. 10.1172/JCI78182 26436651 PMC4639994

[B41] Mordue D. G. Sibley L. D. (2003). A novel population of Gr-1+-activated macrophages induced during acute toxoplasmosis. J. Leukoc. Biol. 74, 1015–1025. 10.1189/jlb.0403164 12972511

[B42] Nathan C. Ding A. (2010). Nonresolving inflammation. Cell 140, 871–882. 10.1016/j.cell.2010.02.029 20303877

[B43] Norling L. V. Sampaio A. L. Cooper D. Perretti M. (2008). Inhibitory control of endothelial galectin-1 on in vitro and in vivo lymphocyte trafficking. FASEB J. 22, 682–690. 10.1096/fj.07-9268com 17965266

[B44] Paclik D. Werner L. Guckelberger O. Wiedenmann B. Sturm A. (2011). Galectins distinctively regulate central monocyte and macrophage function. Cell Immunol. 271, 97–103. 10.1016/j.cellimm.2011.06.003 21724180

[B45] Rabinovich G. A. Croci D. O. (2012). Regulatory circuits mediated by lectin-glycan interactions in autoimmunity and cancer. Immunity 36, 322–335. 10.1016/j.immuni.2012.03.004 22444630

[B46] Rabinovich G. A. Sotomayor C. E. Riera C. M. Bianco I. Correa S. G. (2000). Evidence of a role for galectin-1 in acute inflammation. Eur. J. Immunol. 30, 1331–1339. 10.1002/(SICI)1521-4141(200005)30:5<1331::AID-IMMU1331>3.0.CO;2-H 10820379

[B47] Rostoker R. Yaseen H. Schif-Zuck S. Lichtenstein R. G. Rabinovich G. A. Ariel A. (2013). Galectin-1 induces 12/15-lipoxygenase expression in murine macrophages and favors their conversion toward a pro-resolving phenotype. Prostaglandins Lipid Mediat. 107, 85–94. 10.1016/j.prostaglandins.2013.08.001 23954858

[B48] Ruiz Silva M. Van Der Ende-Metselaar H. Mulder H. L. Smit J. M. Rodenhuis-Zybert I. A. (2016). Mechanism and role of MCP-1 upregulation upon chikungunya virus infection in human peripheral blood mononuclear cells. Sci. Rep. 6, 32288. 10.1038/srep32288 27558873 PMC4997611

[B49] Schif-Zuck S. Gross N. Assi S. Rostoker R. Serhan C. N. Ariel A. (2011). Saturated-efferocytosis generates pro-resolving CD11b low macrophages: modulation by resolvins and glucocorticoids. Eur. J. Immunol. 41, 366–379. 10.1002/eji.201040801 21268007 PMC3082320

[B50] Seropian I. M. Cerliani J. P. Toldo S. Van Tassell B. W. Ilarregui J. M. Gonzalez G. E. (2013). Galectin-1 controls cardiac inflammation and ventricular remodeling during acute myocardial infarction. Am. J. Pathol. 182, 29–40. 10.1016/j.ajpath.2012.09.022 23142379 PMC5691326

[B51] Severa M. Rizzo F. Giacomini E. Salvetti M. Coccia E. M. (2015). IFN-beta and multiple sclerosis: cross-talking of immune cells and integration of immunoregulatory networks. Cytokine Growth Factor Rev. 26, 229–239. 10.1016/j.cytogfr.2014.11.005 25498525

[B52] Snell L. M. Mcgaha T. L. Brooks D. G. (2017). Type I Interferon in Chronic Virus Infection and Cancer. Trends Immunol. 38, 542–557. 10.1016/j.it.2017.05.005 28579323 PMC8059441

[B53] Spite M. Claria J. Serhan C. N. (2014). Resolvins, specialized proresolving lipid mediators, and their potential roles in metabolic diseases. Cell Metab. 19, 21–36. 10.1016/j.cmet.2013.10.006 24239568 PMC3947989

[B54] Starossom S. C. Mascanfroni I. D. Imitola J. Cao L. Raddassi K. Hernandez S. F. (2012). Galectin-1 deactivates classically activated microglia and protects from inflammation-induced neurodegeneration. Immunity 37, 249–263. 10.1016/j.immuni.2012.05.023 22884314 PMC3428471

[B55] Stowell S. R. Qian Y. Karmakar S. Koyama N. S. Dias-Baruffi M. Leffler H. (2008). Differential roles of galectin-1 and galectin-3 in regulating leukocyte viability and cytokine secretion. J. Immunol. 180, 3091–3102. 10.4049/jimmunol.180.5.3091 18292532

[B56] Sugimoto M. A. Vago J. P. Perretti M. Teixeira M. M. (2019). Mediators of the Resolution of the Inflammatory Response. Trends Immunol. 40, 212–227. 10.1016/j.it.2019.01.007 30772190

[B57] Sundblad V. Morosi L. G. Geffner J. R. Rabinovich G. A. (2017). Galectin-1: A Jack-of-All-Trades in the Resolution of Acute and Chronic Inflammation. J. Immunol. 199, 3721–3730. 10.4049/jimmunol.1701172 29158348

[B58] Sundblad V. Quintar A. A. Morosi L. G. Niveloni S. I. Cabanne A. Smecuol E. (2018). Galectins in Intestinal Inflammation: Galectin-1 Expression Delineates Response to Treatment in Celiac Disease Patients. Front. Immunol. 9, 379. 10.3389/fimmu.2018.00379 29545799 PMC5837985

[B59] Tacnet-Delorme P. Gabillet J. Chatfield S. Thieblemont N. Frachet P. Witko-Sarsat V. (2018). Proteinase 3 Interferes With C1q-Mediated Clearance of Apoptotic Cells. Front. Immunol. 9, 818. 10.3389/fimmu.2018.00818 29755460 PMC5932363

[B60] Than N. G. Romero R. Erez O. Weckle A. Tarca A. L. Hotra J. (2008). Emergence of hormonal and redox regulation of galectin-1 in placental mammals: implication in maternal-fetal immune tolerance. Proc. Natl. Acad. Sci. U. S. A 105, 15819–15824. 10.1073/pnas.0807606105 18824694 PMC2556362

[B61] Thieblemont N. Witko-Sarsat V. Ariel A. (2018). Regulation of macrophage activation by proteins expressed on apoptotic neutrophils: Subversion towards autoimmunity by proteinase 3. Eur. J. Clin. Invest. 48 Suppl 2, e12990. 10.1111/eci.12990 30039869

[B62] Toscano M. A. Bianco G. A. Ilarregui J. M. Croci D. O. Correale J. Hernandez J. D. (2007). Differential glycosylation of TH1, TH2 and TH-17 effector cells selectively regulates susceptibility to cell death. Nat. Immunol. 8, 825–834. 10.1038/ni1482 17589510

[B63] Ueha S. Shand F. H. Matsushima K. (2012). Cellular and molecular mechanisms of chronic inflammation-associated organ fibrosis. Front. Immunol. 3, 71. 10.3389/fimmu.2012.00071 22566952 PMC3342381

[B64] Vas V. Fajka-Boja R. Ion G. Dudics V. Monostori E. Uher F. (2005). Biphasic effect of recombinant galectin-1 on the growth and death of early hematopoietic cells. Stem Cells 23, 279–287. 10.1634/stemcells.2004-0084 15671150

[B65] Venkatesh D. Ernandez T. Rosetti F. Batal I. Cullere X. Luscinskas F. W. (2013). Endothelial TNF receptor 2 induces IRF1 transcription factor-dependent interferon-beta autocrine signaling to promote monocyte recruitment. Immunity 38, 1025–1037. 10.1016/j.immuni.2013.01.012 23623383 PMC3760474

[B66] Villa A. Rizzi N. Vegeto E. Ciana P. Maggi A. (2015). Estrogen accelerates the resolution of inflammation in macrophagic cells. Sci. Rep. 5, 15224. 10.1038/srep15224 26477569 PMC4609992

[B67] Voll R. E. Herrmann M. Roth E. A. Stach C. Kalden J. R. Girkontaite I. (1997). Immunosuppressive effects of apoptotic cells. Nature 390, 350–351. 10.1038/37022 9389474

[B68] Wang S. B. Hu K. M. Seamon K. J. Mani V. Chen Y. Gronert K. (2012). Estrogen negatively regulates epithelial wound healing and protective lipid mediator circuits in the cornea. FASEB J. 26, 1506–1516. 10.1096/fj.11-198036 22186873 PMC3316908

[B69] Yamada T. Tani Y. Nakanishi H. Taguchi R. Arita M. Arai H. (2011). Eosinophils promote resolution of acute peritonitis by producing proresolving mediators in mice. FASEB J. 25, 561–568. 10.1096/fj.10-170027 20959515

